# Pathologic manifestations of levamisole-adulterated cocaine exposure

**DOI:** 10.1186/s13000-015-0279-z

**Published:** 2015-05-06

**Authors:** Amber L Nolan, Kuang-Yu Jen

**Affiliations:** Department of Pathology, University of California San Francisco, 513 Parnassus Avenue, Box 0102, San Francisco, CA 94143 USA

**Keywords:** Anti-neutrophil cytoplasmic antibody, Pauci-immune, Glomerulonephritis, Leukocytoclastic, Vasculitis, Levamisole, Cocaine

## Abstract

**ᅟ:**

Rheumatic manifestations of cocaine have been well described, but more recently, a dramatic increase in the levamisole-adulterated cocaine supply in the United States has disclosed unique pathologic consequences that are distinct from pure cocaine use. Most notably, patients show skin lesions and renal dysfunction in the setting of extremely high perinuclear anti-neutrophil cytoplasmic antibodies (p-ANCA). Unexpectedly, antibodies to myeloperoxidase, the typical target of p-ANCA, are relatively low if at all present. This discrepancy is due to the fact that p-ANCA seen in association with levamisole-adulterated cocaine exposure is often directed against atypical p-ANCA-associated antigens within the neutrophil granules such as human neutrophil elastase, lactoferrin, and cathepsin G. Biopsies of the skin lesions reveal leukocytoclastic vasculitis often involving both superficial and deep dermal vessels. Renal injury most typically manifests as crescentic and necrotizing pauci-immune glomerulonephritis. In this review, the manifestations of levamisole-adulterated cocaine-induced vasculitis are discussed with an emphasis on the typical histomorphologic findings seen on biopsy.

**Virtual Slides:**

The virtual slide(s) for this article can be found here: http://www.diagnosticpathology.diagnomx.eu/vs/1764738711370019.

## Introduction

Rheumatic consequences of cocaine are rare but well documented. These disorders primarily manifest as organ specific vasculitis (such as cerebral vasculitis), systemic vasculitis, or cocaine-induced midline destructive lesions (CIMDL) [1]. In the case of cocaine-induced systemic vasculitis, the clinical and laboratory findings are essentially indistinguishable from primary, idiopathic granulomatosis with polyangiitis (GPA), which is characterized by skin lesions, nasal and palate destruction, pauci-immune glomerulonephritis with crescents and/or necrotizing lesions, and positive laboratory test for cytoplasmic antineutrophil cytoplasmic antibodies (c-ANCA) associated with proteinase-3 (PR3) antibodies [2-4]. CIMDL can closely mimic the upper airway lesions seen in GPA with reports ranging from severe sinusitis to nasal and palatal perforation; however, the pathophysiology is thought to be secondary to chronic cocaine exposure, leading to progressive mucosal and perichondrial injury and subsequent ischemic necrosis and perforation of the nasal septum [5,6]. Histologically, CIMDL is distinguished from GPA by the lack of granulomatous inflammation and vasculitis on biopsy, and serologic differences have been reported [7,5,8,9]. While these pathologies are rare, it is important to recognize the association with cocaine use, as cessation of the drug is often the only treatment needed.

Since 2005, a new vasculitic syndrome appears to be occurring with increasing frequency in patients who use cocaine, characterized by positive perinuclear (p)-ANCA serologies, neutropenia/agranulocytosis, and purpuric lesions involving the face, especially the ear lobes, with varying frequencies of glomerulonephritis and lung hemorrhage. The increased prevalence appears to be correlated with the escalating abuse of levamisole-adulterated cocaine [1]. This review will summarize the range of clinical and pathologic findings seen in the setting of levamisole-adulterated cocaine exposure with an emphasis on the morphologic features observed in patient biopsies.

## Review

### Levamisole and levamisole-adulterated cocaine

Levamisole, a synthetic imidazothiazole derivative, is an anti-helminthic drug with immunomodulatory activity, which has been used in the past to treat a variety of conditions including malignancy, autoimmune disorders such as rheumatoid arthritis, and pediatric nephrotic syndrome [10-12]. It was removed from the United States market for human use in 1999 due to significant side effects including agranulocytosis and thrombocytopenia [13-15]. Chronic use of levamisole is associated with positive ANCA serologies and a characteristic skin rash of retiform purpura involving the ears, face, and extremities. Accompanying skin biopsies often show leukocytoclastic vasculitis [16,17].

Levamisole-adulterated cocaine has been increasing in frequency in the United States drug supply since 2005 with a prevalence now estimated at up to 70-80% of the cocaine entering the country [18-20]. It has not been clearly elucidated as to why this compound has become such a common additive in cocaine, but several explanations have been proposed. Levamisole may inhibit monoamine oxidase and catechol-O-methyltransferase, potentiating the level of dopamine in the central nervous system and leading to a higher activation of the reward system [21,22]. Furthermore, in the muscle cells of helminthes, this drug blocks nicotinic acetylcholine receptors; a similar action in humans may have a secondary effect on the release of dopamine [22,21]. A metabolite of levamisole, aminorex, could have amphetamine-like stimulant properties as well, or may act indirectly as a serotonin receptor agonist [21,13]. Alternatively, levamisole may purely be used as a cutting agent to add volume while maintaining a white powdery consistency [21]. Regardless of the reason, increasing levamisole-associated pathology is being identified across the country and prompt recognition of a few characteristic features may help guide treatment and prevent further long-term complications.

### Signs and symptoms

Patients with symptoms of levamisole-associated vasculitis due to cocaine exposure often experience nonspecific constitutional symptoms such as fever, fatigue, flu-like symptoms, night sweats, and weight loss. Arthralgia and cutaneous manifestations are also quite common clinical findings and often motivate the individual to seek medical care [23]. Additionally, extra-cutaneous involvement may be present (detailed below).

### Anti-neutrophil cytoplasmic antibodies and other serologies

Patients with vasculitis associated with levamisole-adulterated cocaine classically demonstrate unique serologic abnormalities characterized by unusually high titers of p-ANCA without substantial antibodies against myeloperoxidase (MPO), the typical target of p-ANCA. This conflicting finding is due to high titer p-ANCA production that is often directed against atypical p-ANCA-associated antigens within the neutrophil granules such as human neutrophil elastase (HNE), lactoferrin, and cathepsin G [1,24]. Such serologic findings are characteristic of levamisole-associated autoimmune disease but not specific to this entity since similar serologic profiles can be encountered in other drug-induced ANCA-associated vasculitides [25].

Interestingly, some p-ANCA-positive patients with levamisole-adulterated cocaine exposure may show the presence of anti-PR3 antibodies, which typically are c-ANCA. Some speculate that this discordant observation may be due to cross-reactivity of the antibodies against homologous epitopes on PR3 and HNE [1]. In addition, the majority of cases are also positive for antiphospholipid antibodies such as lupus anticoagulant and anti-cardiolipin antibody as well as antinuclear and anti-double-stranded DNA antibodies [24]. These features can help distinguish levamisole-adulterated cocaine-induced pathology from idiopathic ANCA-associated vasculitis, which may present with a similar clinical picture but would demonstrate lower ANCA titers with specific antibodies to one neutrophilic antigen rather than several.

Pure cocaine use can also provoke ANCA-associated vasculitis. However, cocaine-induced GPA demonstrates positive c-ANCA and PR3 antibody in contrast to the p-ANCA predominant pattern seen with levamisole [2]. Antiphospholipid antibodies have not been reported in patients with pure cocaine-associated autoimmune disease [26,24].

### Hematological abnormalities

Levamisole-adulterated cocaine was originally brought to clinical attention after several case reports of severe agranulocytosis associated with cocaine use emerged in a clinical reference laboratory in New Mexico [7,20]. Morphologic analysis of the bone marrow and blood demonstrated increased plasmacytoid lymphocytes, bone marrow plasmacytosis, and megakaryocyte hyperplasia with increased frequency in the cocaine-exposed agranulocytosis group [20]. In small studies, neutropenia was reported with an incidence of 4.2% in cocaine users in general and an incidence of 2.1% in users with documented exposure to levamisole and cocaine [27]. In a retrospective analysis investigating patients with exposure to cocaine in addition to high p-ANCA titers or combined positivity for MPO and PR3 antibodies, 28% had leukopenia, of which half had an absolute neutrophil count of <1000 [23]. These findings are compatible with the toxicity levels seen with levamisole use alone where 2.5-13% of patients developed neutropenia or agranulocytosis, which occurs in a dose-dependent fashion [14,28,29,15].

Interestingly, neutropenia/agranulocytosis is a feature not commonly associated with pure cocaine-linked rheumatologic disease, and is not reported as a general characteristic of a drug-induced ANCA-associated vasculitis [30]. Thus, this hematologic abnormality may be a clue to suspect levamisole-induced pathology.

### Skin pathology

Another distinguishing finding of levamisole-adulterated cocaine-induced pathology is characteristic skin lesions, which often involve the ear lobes and the skin overlying the zygomatic arch [24]. Other frequent areas of involvement include the extremities, particularly the lower extremities, with relative sparing of the trunk and back [31,24,32]. The rash often presents as purpuric plaques with a retiform pattern in half of patients and central necrosis in a third of patients [31]. In individuals with skin lesions, associated hematological abnormalities occur in approximately 60%, while 95-100% of patients have positive ANCA serologies [24,31].

**Figure 1:**
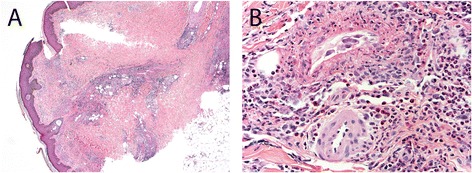
**Skin biopsy from a patient with exposure to levamisole-adulterated cocaine.** **A)** Leukocytoclastic vasculitis involves both superficial and deep dermal vessels (hematoxylin and eosin stain, 40X). **B)** Numerous neutrophils and eosinophils surround and involve the vascular walls associated with leukocytoclastic debris and fibrinoid necrosis (hematoxylin and eosin stain, 400X).

Biopsies of the skin lesions generally show small vessel vasculitis in the form of leukocytoclastic vasculitis, with or without intravascular thrombi [31,33,24,32] (Figure 1). Typically, both superficial and deep small dermal vessels are involved by angiocentric mixed inflammatory cell infiltrates prominent in neutrophils, and at times also frequent in eosinophils. The inflammation infiltrates the vascular walls and into the perivascular zones and usually shows prominent leukocytoclastic debris (nuclear dust). Fibrinoid necrosis of vascular walls can be seen and often extends into the adjacent perivascular connective tissue. Extravasation of red blood cells is also a common histologic finding. Direct immunofluorescence may highlight antibody and complement deposition in vessel walls [31]. These findings are similar to reports from chronic levamisole use in pediatric patients treated for nephrotic syndrome; in one case series, 5 of 160 children developed purpura on at least the ears with biopsies revealing cutaneous vasculitis or thrombotic lesions [17]. The cutaneous lesions observed in ANCA-associated systemic vasculitis typically demonstrate similar histopathology of leukocytoclastic vasculitis; however, the predilection for the ears and zygomatic arch appears to represent a discriminating feature with levamisole-adulterated cocaine exposure [24,30].

### Renal pathology

Despite sharing many features with other drug-induced ANCA-associated vasculitides, organ involvement is not commonly discussed with levamisole-adulterated cocaine-induced autoimmune disease. However, recent case reports suggest that this aspect may have been overlooked. In a retrospective analysis investigating patients with unusually high p-ANCA titers and exposure to cocaine, eight out of thirty patients were reported to have proteinuria or hematuria, and two developed acute kidney injury [23]. One patient underwent renal biopsy showing focal necrotizing and crescentic pauci-immune glomerulonephritis. In another case report, a 45-year-old man presented with characteristic necrotic skin lesions and an associated positive ANCA serology as well as a positive toxicology screen for cocaine and levamisole on mass spectrometry. He displayed both hematuria and proteinuria, was treated with prednisone, and left against medical advice. Seven months later, he presented after repeat cocaine use with acute on chronic renal insufficiency and a renal biopsy displaying necrotizing and crescentic pauci-immune glomerulonephritis [34].

**Figure 2:**
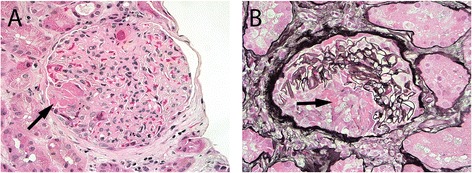
**Kidney biopsy from a patient with exposure to levamisole-adulterated cocaine.** **A)** Necrotizing lesion characterized by the presence of fibrin (arrow) within the Bowman space with associated nuclear debris and incipient cellular crescent (hematoxylin and eosin stain, 400X). **B)** Cellular crescent with fibrin (arrow) within the Bowman space (methenamine silver-Periodic acid-Schiff stain, 400X). Note that the glomerular tuft is otherwise relatively normal, as is often seen in pauci-immune glomerulonephritis.

In a review of an inner city hospital’s pauci-immune glomerulonephritis cases over a two-year period, four patients were identified with disease who had exposure to cocaine, serologies compatible with levamisole exposure, and one other common clinical indicator of levamisole exposure (purpura, digital infarcts, or neutropenia) [35]. These biopsies demonstrated a range of glomerular crescents from cellular to fibrocellular to fibrous crescents, reflecting the relative timeframe of levamisole-adulterated cocaine exposure in relation to time of biopsy. Cases with cellular crescents also had associated necrotizing lesions (Figure 2). Patchy acute tubular epithelial cell injury was commonly noted, and one case with mostly fibrous/fibrocellular crescents exhibited significant interstitial fibrosis and tubular atrophy. Vessels showed the typical chronic changes seen in association with age and drug use, but no evidence of malignant hypertension or thrombotic microangiopathy was detected. Immunofluorescence stains for immunoglobulins (IgG, IgM, and IgA) and complement components (C3 and C1q) were negative in all cases. Electron microscopy confirmed the lack of immune complex deposition. Thus, these features were classified as crescentic and necrotizing pauci-immune glomerulonephritis.

These reports suggest a role for levamisole-adulterated cocaine in the induction of pauci-immune glomerulonephritis. In the kidney, pure cocaine use without an autoimmune syndrome most commonly leads to severe intimal thickening of arteries reflecting accelerated arteriosclerosis. Rare cases of thrombotic microangiopathy due to malignant hypertension and renal infarction have been reported [36-38]. Cocaine-induced GPA with a positive c-ANCA serology can be associated with a pauci-immune glomerulonephritis [2,4,3]. Interestingly, glomerulonephritis is not a reported complication of levamisole-use alone, and this pathology may represent a synergistic effect of cocaine and levamisole.

### Pulmonary pathology

As with other drug-induced vasculitides, which are known to involve a triad of skin, kidney and lung, pulmonary pathology is occasionally reported with levamisole-adulterated cocaine exposure. Diffuse alveolar hemorrhage, requiring intubation, was reported in one patient who also had concurrent pauci-immune glomerulonephritis [35]. Three out of thirty patients with cocaine exposure and high p-ANCA antibodies developed pulmonary hemorrhage not requiring intubation; none of these patients had convincing coexisting renal disease [23]. Aminorex, a metabolite of levamisole, has been associated with one case of idiopathic pulmonary hypertension [39]. No biopsies have been reported to further characterize this associated lung pathology, and no specific upper airway lesions have been identified or discussed with this syndrome.

### Mechanisms of levamisole-adulterated cocaine-induced pathology

The mechanism whereby levamisole-adulterated cocaine leads to vasculitis, glomerulonephritis, and agranulocytosis is unclear, but several theories have been proposed. As with other drug-induced ANCA-associated vasculitides, the mechanism is hypothesized to be a direct consequence of the anti-neutrophilic antibodies. Several clinical observations support this theory in typical ANCA-associated vasculitis. First, antibody titers appear to correlate with disease relapse [40,41]. Furthermore, treatment with B-cell depleting rituximab successfully treats symptoms of ANCA-associated disease [30]. Additionally, transplacental MPO-ANCA was directly associated with kidney and lung disease in a neonate, which resolved shortly after birth [42,43]. Animal models also exhibit pauci-immune glomerulonephritis and small vessel vasculitis after development of antibodies to MPO. The proposed mechanism supports that priming of neutrophils leads to translocation of ANCA antigens to the cell surface. In the presence of ANCA, such antigens lead to further neutrophil activation and increased adherence and migration of neutrophils through the endothelium. In addition, release of reactive oxidative species and neutrophil degranulation causes small vessel destruction [30]. T-cell-mediated immunity is also critical to disease development. In mouse models, T-cell depletion inhibits vasculitis, and a MPO T-cell epitope is essential for glomerular injury [44,30].

How these antibodies develop is also under much investigation. A predisposition to autoimmune disease is associated with levamisole-induced pathology, as there appears to be an increased occurrence in women, those with rheumatic disease, and patients carrying the HLA B27 genotype [20]. Levamisole may interact with neutrophil extracellular traps (NETs), which are composed of a complex of DNA, histones and neutrophil granules including MPO, PR3, and HNE. NETs could be released in response to stress and provide a source of antigen that could activate the immune system. Certain medications, such as propylthiouracil, are correlated with disorganized NET structure, promoting prolonged degradation and possible antigenicity [45]. Some drugs may also accumulate in neutrophils and bind to MPO, causing production of a subsequent auto-antigen; drug-induced neutrophil apoptosis may also overstimulate the immune system [30].

The reactive electrophile has specifically been put forth as a mechanism for drug-mediated agranulocytosis. More specifically, it has been hypothesized that an electrophilic reactive metabolite of levamisole may act as a hapten and react with a self-peptide to generate an immune response. Subsequently, T-cell activation occurs and results in a delayed hypersensitivity reaction, which fits with the clinical timeframe for levamisole-induced agranulocytosis [46]. A similar hypothesis has also been described for other drugs, such a propylthiouracil, with side effects including agranulocytosis and drug-induced vasculitis.

## Conclusion

In summary, levamisole-adulterated cocaine use is a growing public health concern. It is associated with an autoimmune syndrome that typically presents with non-specific flu-like symptoms, neutropenia/agranulocytosis, high titers of several p-ANCA antibodies, and a characteristic rash of retiform purpura with possible necrosis involving the ears, face and extremities. Organ involvement also appears to be possible with several case reports of both renal and lung disease that is most commonly expressed as pauci-immune glomerulonephritis and pulmonary hemorrhage, respectively. The mechanism of disease is still under investigation but hinges on drug-induced production of auto-antibodies. Treatment depends on the organ systems involved. Patients with this condition may require less immunosuppressive medication compared to idiopathic vasculitides since cessation of drug use appears to lead to drastic improvements.

## Abbreviations

GPA: Granulomatosis with polyangiitis
CIMDL: Cocaine-induced midline destructive lesions
c-ANCA: Cytoplasmic antineutrophil cytoplasmic antibodies
p-ANCA: Perinuclear anti-neutrophil cytoplasmic antibodies
PR-3: Proteinase-3
NETs: Neutrophil extracellular traps
